# Breast ultrasound: automated or hand-held? Exploring patients’ experience and preference

**DOI:** 10.1186/s41747-019-0136-z

**Published:** 2020-02-10

**Authors:** Ilaria Mussetto, Licia Gristina, Simone Schiaffino, Simona Tosto, Edoardo Raviola, Massimo Calabrese

**Affiliations:** 1 School of Radiology, University of Genoa, Department of Health Sciences DISSAL, Via Antonio Pastore 1, 16132 Genoa, Italy; 2 Diagnostic Senology, IRCCS – Policlinico San Martino, Largo Rosanna Benzi 10, 16132 Genoa, Italy; 3 Radiology Unit, IRCCS Policlinico San Donato, Via Morandi 30, San Donato Milanese, 20097 Milan, Italy; 4 Università Vita-Salute, San Raffaele, Via Olgettina 58, 20132 Milan, Italy

**Keywords:** Anxiety, Fear, Pain, Personal satisfaction, Ultrasonography (mammary)

## Abstract

**Background:**

Our aim was to compare women’s experience with automated breast ultrasound (ABUS) *versus* breast hand-held ultrasound (HHUS) and to evaluate their acceptance rate.

**Methods:**

After ethical approval, from October 2017 to March 2018, 79 consecutive patients were enrolled in this prospective study. On the same day, patients underwent HHUS followed by ABUS. Each patient’s experience was assessed using the modified testing morbidities index (TMI) (the lower the score, the better is the experience). Nine items were assessed for both techniques: seven directly related to the examination technique (pain or discomfort immediately before (preparation), during and after testing, fear or anxiety immediately before (preparation) and during testing, physical and mental function after testing) and two indirectly related to the examination technique (embarrassment during testing and overall satisfaction). Finally, we asked patients to choose between the two techniques for a potential next breast examination. Wilcoxon signed ranks test was used.

**Results:**

The median TMI score for the seven items was found to be significantly better for HHUS (8, interquartile range [IQR] 7–11) compared to ABUS (9, IQR 8–12) (*p* = 0.003). The item ‘pain/discomfort during the test’ (*p* < 0.001) was significantly higher for ABUS compared to HHUS. Instead, the item ‘fear/anxiety before the test’ was higher for HHUS (*p* = 0.001). Overall, 40.5% of the patients chose HHUS, 29.1% chose ABUS, and 30.4% were unable to choose.

**Conclusions:**

ABUS and HHUS exams were well tolerated and accepted. However, HHUS was perceived to be less painful than ABUS.

## Key points

- Patient’s experience of automated breast ultrasound and hand-held breast ultrasound was assessed using the modified testing morbidities index.
- Both examinations were well tolerated.
- Hand-held breast ultrasound was perceived as less painful and more comfortable than automated breast ultrasound.

## Background

Automated breast ultrasound (ABUS) is a volumetric sonographic technique in which the whole breast volume is acquired with almost isotropic voxels, providing multiplanar reconstruction of the breast [1, 2]. The main advantage of ABUS over the usual hand-held whole-breast ultrasound (HHUS) is the standardised acquisition with a decrease in both operator dependency and physician workload. With this technique, the time of image acquisition is separated from image interpretation [3]. The ABUS system has a standardised acquisition protocol that can be performed, after a short period of training, by the radiographer or the sonographer, based on different countries. Interpretation is performed by a breast radiologist on dedicated workstations with mean reading times reported in the literature from less than 3 to 10 min [4–6].

Most research that has been done with ABUS showed promising results in the screening setting, especially in women with dense breast tissue, as shown by Wilczek et al. [7]. Recently, other studies have also evaluated ABUS in the diagnostic setting such as breast cancer staging, neoadjuvant therapy response evaluation, and second look after magnetic resonance imaging [8]. However, there are only a few works which evaluated patient experience with ABUS compared to HHUS [9].

Thus, our aim was to compare the women’s experience with ABUS and HHUS and to evaluate the acceptance rate of these two examinations used and intra-individual design.

## Methods

### Participant recruitment

From October 2017 to March 2018, consecutive patients referring to our academic medical centre for breast HHUS were asked to participate in this study. All consecutive females undergoing breast ultrasound were invited to undergo ABUS as an optional examination, regardless of the clinical indication for examination: adjunct screening tool in women with dense breast, follow-up of known benign breast lesions or preoperative assessment of histologically proven lesions.

The Ethics Committee of our hospital approved the research plan of the study on June 29, 2017. Informed written consent was obtained from all participants included in the study.

On the same day, patients who agreed to participate in the study underwent HHUS (iU22, Philips, Eindhoven, Netherlands or MyLab70 XVG, Esaote, Genoa, Italy) followed by ABUS examination (Invenia ABUS, GE Healthcare, Milwaukee, WI, USA). HHUS examinations were performed by one of the three breast dedicated radiologists with over 10 years of experience in breast ultrasound. HHUS includes the evaluation of the whole breast parenchyma bilaterally.

ABUS was performed by a trained radiographer and reviewed by one of the three breast dedicated radiologists that performed the HHUS.

ABUS examination was carried out with the patient lying in a supine position, with the breast flattened by the compression of a reverse curve transducer. Immediately before the acquisition, the probe was positioned on the breast (preparation phase) and then the probe moved to collect the images (acquisition phase). At least three views were acquired for each breast (anteroposterior, lateral, and medial), with an acquisition time of about 60 s each, with a total examination time of about 15 min.

### Data collection

Immediately after the execution of each examination (HHUS or ABUS), the patient’s experience was assessed using the modified testing morbidities index (TMI) for both techniques, with a self-administered questionnaire. TMI was obtained before giving the results to patients to avoid biases related to the results of the examinations. According to previous works [10–12], TMI is a validated instrument for the assessment of short-term life quality related to diagnostic testing. It was slightly modified for the purpose of our study. Nine attributes were assessed for both techniques: (a) pain or discomfort immediately before the test, (b) pain or discomfort during the test, (c) pain or discomfort after the test, (d) fear or anxiety immediately before the test, (e) fear or anxiety during the test, (f) physical function after testing, (g) mental function after testing, (h) embarrassment during the test, and (i) overall satisfaction.

The first seven attributes (a–g) were directly related to the examination, while the remaining two (h and i) were only indirectly related (or not necessarily related) to the examination, as already hypothesised by Tagliafico et al. [10] for the attribute (h).

Patients used a 5-point scale assessment for the seven attributes related to the examination (a–g) and to describe the level of embarrassment during the test, where 1, none; 2, mild; 3, moderate; 4, severe; and 5, extreme. Regarding physical and mental function after testing, the following scale was used: 1, no problems; 2, mild problems; 3, moderate problems; 4, severe problems; and 5, extreme problems. The questionnaire also included questions about the patient’s overall satisfaction, such as ‘the doctor explained what to expect during the exam’ and ‘the staff showed concern for my worries’. Patients assessed their overall satisfaction (i) by means of the following 4-point scale: 1, strongly agree; 2, somewhat agree; 3, somewhat disagree; and 4, strongly disagree. The lower is the given score, the better is the patient’s experience with the ultrasound examination.

The last question we asked the patients was to choose between ABUS and HHUS for a potential next breast examination.

Collected demographic and clinical data included age, personal history of breast pathology or previous breast biopsies and family history of breast cancer. Familiarity history and personal history were assessed as follows: 0, none, and 1, yes.

In order to compare the acceptance rate of these two ultrasound examinations, we considered the seven attributes strictly related to the examination (a–g); therefore, the TMI score of these seven items, for each patient, ranged from 7 (best possible experience) to 35 (worst possible experience).

### Statistical analysis

Wilcoxon signed ranks test was used to compare categorical variables of ABUS and HHUS. Univariate and multivariate linear regression analyses were conducted to identify whether age, personal history or family history was an independent predictor of the TMI score. Age was reported as mean ± standard deviation, taking into consideration normal distribution, confirmed by D’Agostino-Pearson test (*p* = 0.881).

Statistical analysis was performed with SPSS software (IBM Corp., New York, NY; formerly SPSS Inc., Chicago, IL, United States). We considered *p* values lower than 0.050 as statistically significant to avoid the introduction of type I errors. Bonferroni’s correction was applied for multiple comparisons to the preset level of significance; the significance level was therefore *p* < 0.006 (0.050/9).

## Results

Of 92 patients invited to perform the additional ABUS examination, 79 (85.9%), all females, accepted and were enrolled in the study, with a mean age of 53 ± 14 years (range 18–87 years). Table 1 shows patient characteristics. Of 79 enrolled patients, 74 (93.7%) completed the ABUS examination, while 5 (6.3%) interrupted the execution due to experiencing discomfort during the test.

**Table 1:** Patient characteristics

Patients Characteristics	No. of patients (*n* = 79)
Age (years)	
≤ 50	43 (54.4)
51–59	14 (17.7)
≥ 60	22 (27.8)
Family history of breast cancer	
No	48 (60.8)
Yes	31 (39.2)
Personal history of breast pathology or previous biopsy	
No	32 (40.5)
Yes	47 (59.5)

Data are number of patients with percentages in parentheses

The median TMI score for the seven examination-related items was low in both techniques: 9 (IQR 8–12) for ABUS and 8 (IQR 7–11) for HHUS. At Wilcoxon signed ranks test for related samples, the TMI score was significantly better for HHUS compared to ABUS (*p* = 0.003).

The ABUS technique received higher total scores in each of the attributes related to the examination except for item (d) ‘fear or anxiety immediately before the test’, which was significantly higher for HHUS (*p* = 0.001). Regarding the attribute (b), pain or discomfort during the test, it was significantly higher for ABUS at Wilcoxon signed ranks test, with a median value for ABUS and HHUS of 3 (IQR 2–3) and 1 (IQR 1–2), respectively (*p* < 0.001).

The level of embarrassment during testing and the overall satisfaction was similar for both the procedures and no significant difference was found when comparing the two (*p* ≥ 0.170).

With univariate and multivariate linear regression analysis, neither age, personal history nor family history was found to be a significant independent predictor of the TMI score (*p* ≥ 0.400) for both ABUS and HHUS.

To the last question ‘Which technique would you choose for a potential next breast examination?’, 32/79 (40.5%) patients answered HHUS, 23/79 (29.1%) patients answered ABUS and 24/79 (30.4%) patients did not declared any preference between the two techniques.

**Fig 1:**
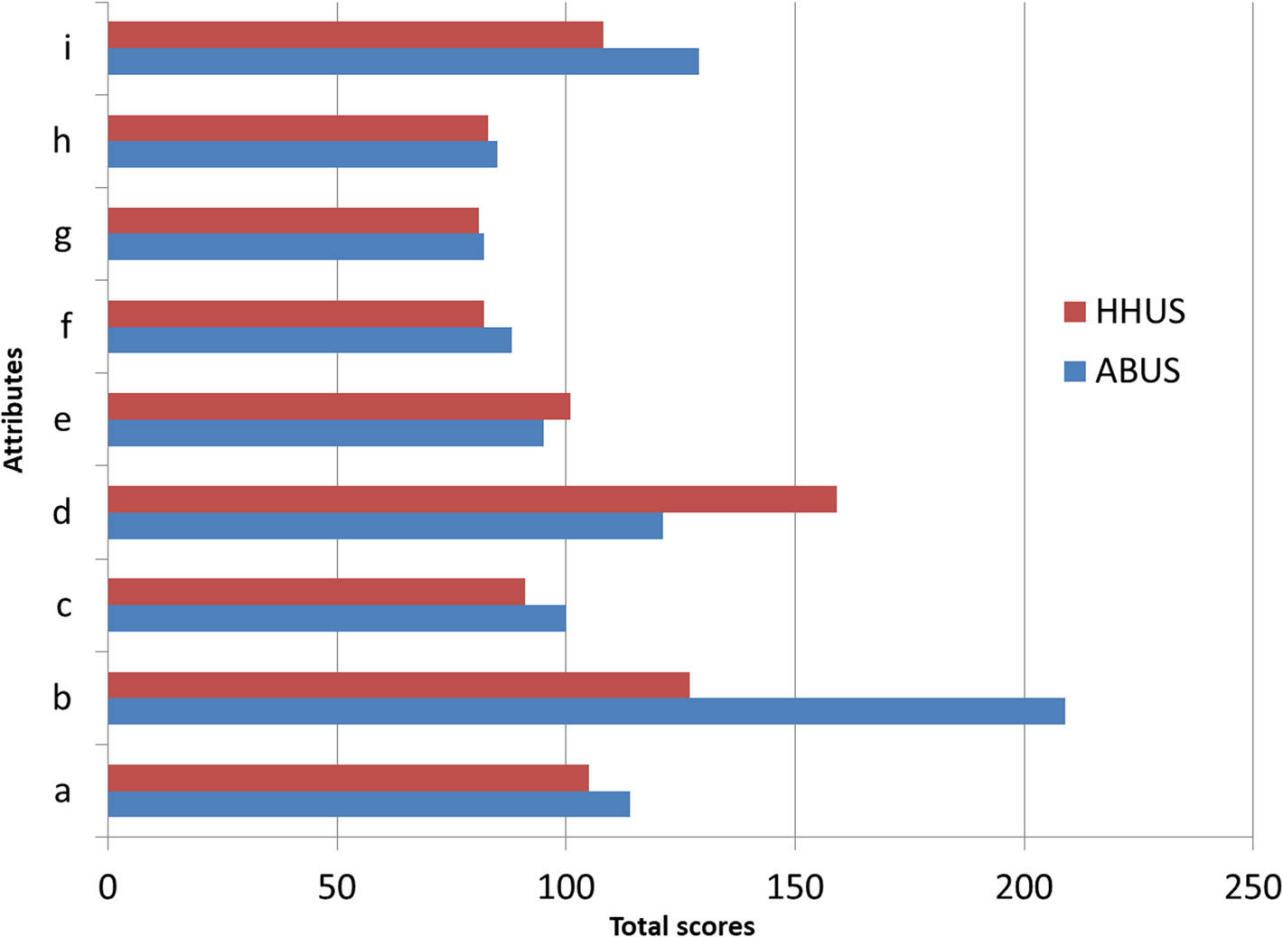
Figure shows the total score for each attribute evaluated both for hand-held ultrasound (HHUS) and automated breast ultrasound (ABUS): (**a**) pain or discomfort immediately before the test, (**b**) pain or discomfort during the test, (**c**) pain or discomfort after the test, (**d**) fear or anxiety immediately before the test, (**e**) fear or anxiety during the test, (**f**) physical function after testing, (**g**) mental function after testing, (**h**) embarrassment during the test, and (**i**) overall satisfaction

Table 2 shows the median values and IQRs for the nine attributes evaluated for each of the two breast ultrasound examinations. Figure 1 shows the total score for each of the two breast ultrasound examinations.

**Table 2:** Median values and interquartile range for the attributes evaluated

Attributes	Median		Interquartile range		*p* value
	ABUS	HHUS	ABUS	HHUS	
Pain or discomfort before the test	1	1	1–2	1–1	*p* = 0.020
Pain or discomfort during the test	3	1	2–3	1–2	*p* < 0.001
Pain or discomfort after the test	1	1	1–1	1–1	*p* = 0.131
Fear or anxiety before the test	1	1	1–1	1–2	*p* = 0.001
Fear or anxiety during the test	1	1	1–1	1–1	*p* = 0.437
Physical function after testing	1	1	1–1	1–1	*p* = 0.107
Mental function after testing	1	1	1–1	1–1	*p* = 0.564
Embarrassment during the test	1	1	1–1	1–1	*p* = 0.577
Overall satisfaction	1	1	1–2	1–2	*p* = 0.060

*ABUS* Automated breast ultrasound, *HHUS* Hand-held ultrasound. Wilcoxon signed ranks test was used. Based on Bonferroni’s correction, *p* < 0.006 (0.050/9) was considered as statistically significant

## Discussion

Automated breast ultrasound is an increasingly used technique for breast cancer diagnosis and appears to be a promising adjunct method to mammography in screening programmes in women with dense breasts [7, 13, 14]. One of the principles required in a screening programme is that the test should be acceptable by the population, as it is important that the patient is comfortable in order to ensure compliance and completion of the exam. In this regard, many studies previously investigated the effect on quality of life of screening mammogram programmes, and false positives have been shown to increase patient anxiety in the short term, to increase patient worries about potentially having breast cancer, which leads to a temporary reduction in the patient’s quality of life [15, 16] .

Previous works [14, 17, 18] have compared ABUS to HHUS in terms of clinical performance in the detection and characterisation of breast lesions, but very few studies [9, 19] evaluated the patient’s perspective and tolerability of the examination.

In our study, the TMI was used to assess the acceptance rate of breast ultrasound comparing ABUS to HHUS. Even though HHUS had better tolerance compared to ABUS, we found that both methods are tolerated by the patients and both could potentially be integrated as adjunct screening tools to mammography.

The main disadvantage of ABUS was the pain or discomfort experienced by individuals during the test. This was expected since breast compression of a large part of the breast is necessary in order to obtain high quality images. Compression is performed both during the preparation phase (the probe is positioned on the breast) and during the acquisition phase (the probe moves to collect the images) which may cause further discomfort. Unfortunately, the technician can only slightly adjust the degree of compression; otherwise, examinations can be unreliable. As mentioned previously, five patients requested to interrupt the examination due to the severe discomfort despite technologist attempts to relieve probe pressure over the patient’s breasts. Nevertheless, we underline that test interruption due to severe discomfort happened in a relatively small percentage of patients, *i.e.*, 6.3% (5/79).

In a previous study, Prosch et al. [9] assessed patient comfort using a standardised questionnaire administered to 76 women undergoing ABUS and HHUS. The ABUS examination was rated as completely painless by 64% patients, 25% indicated minor pain and 10% indicated moderate pain. The HHUS was rated completely painless in 66%, 26% indicated minor pain and 8% indicated moderate pain. However, Zintsmaster et al. [19] demonstrated that ABUS is perceived to be significantly less painful than digital screening mammography.

When considering the question ‘Which technique would you choose for a potential next breast examination?’, 40.5% of patients chose HHUS. This is most likely because many of our patients had already received one or more breast ultrasound examination in the past and are accustomed to the HHUS technique. Another possible factor that favoured HHUS is that it is performed by a medical doctor, mainly a breast radiologist, as it happens in our country, not by a radiographer or sonographer. As a result, the patients have direct contact with the physician who can answer their questions and concerns in real time, which provides more reassurance to the patient during the exam. However, in other countries, HHUS can be performed by radiographers/sonographers, and patients are used to refer directly to radiographers like in a screening mammography acquisition setting. We note that almost one third of patients did not declare a preference for one of the two methods for a potential next breast examination. This result can be interpreted as follows: they had a similar experience and consider the two examinations as interchangeable.

In our study, we did not find a significant association between TMI scores and age, previous personal history or family history of breast cancer, and, in particular, we found no relationship between age and ‘pain or discomfort during the test’. Our results differ from a previous study in which Tagliafico et al. [10] found that age was a significant predictor of short-term quality of life related to breast biopsy.

A potential bias of our study is that all women were subjected to HHUS before the ABUS examination. Due to the close timing between the two exams, it is possible that patient tiredness could have led to a decreased compliance and pain threshold, potentially raising the scores related to ABUS. Furthermore, patients had experienced more fear or anxiety prior to HHUS than ABUS. This could perhaps be due to the fact that patients considered HHUS as the ‘definitive’ diagnostic examination, while ABUS could have been perceived as just an experimental additional test.

Other study limitations were as follows: it represented the experience of a single academic institution; the sample size was relatively small; ABUS was offered to the patients as an optional test and was not part of a daily clinical practice. Finally, we should consider the heterogeneity of the study population: the enrolled women referred for HHUS had several different clinical indications. This can be regarded as a limitation since ABUS is most useful for screening purposes, in particular in women with radiologically dense breasts. In this study, that setting was not emulated. On the other hand, the multiplicity of clinical indications of the study population contributes to a varied intra-individual design and assesses patient’s experience not only in the screening setting but also in the clinical setting.

In conclusion, our findings indicate that both ABUS and HHUS are well tolerated by patients. The HHUS is preferred in a significant fraction of the enrolled patients with the main limitation of ABUS being low tolerance to breast compression.

Further refinements in the probe architecture may improve patient’s tolerance and allow for the use of this new sonographic technique in large populations.

## Abbreviations

ABUS: Automated breast ultrasound
HHUS: Hand-held ultrasound
IQR: Interquartile range
TMI: Testing morbidities index
